# “Whose Fault Is It?” How Rural Chinese Women Explain Intimate Partner Violence: A Qualitative Study

**DOI:** 10.3389/fpsyt.2021.711819

**Published:** 2021-12-02

**Authors:** Fengsu Hou, Catherine Cerulli, Marsha N. Wittink, Eric D. Caine, Peiyuan Qiu

**Affiliations:** ^1^Department of Public Health, Shenzhen Kangning Hospital, Shenzhen, China; ^2^Department of Psychiatry, Medical Center, University of Rochester, Rochester, NY, United States; ^3^Department of Epidemiology and Health Statistics, West China School of Public Health, Sichuan University, Chengdu, China

**Keywords:** intimate partner violence (IPV), explanatory model, women's voices, rural China, social ecological model (SEM)

## Abstract

Women are often the victims of intimate partner violence (IPV). Though China has established its first statute against domestic violence, the service developments for victims fall behind. It is important to assess community members' perceptions of what causes IPV to create interventions to prevent and address IPV. This study completed the Short Explanatory Model Interview (SEMI) among a subset sample from a large epidemiology study in rural Sichuan China. The social ecological model was applied to analyze qualitative interviews. Among 339 participants, the average age was 46.01 ± 12.42 years old. There were 31.86% of them had been educated, 14.75% of them had migrant worker partners, and 49.26% of them had experienced violence from their partners in the last year. There were 252 participants attributed IPV to individual factors, and they primarily discussed the social characteristics, behaviors, personalities or even health problems of the husband or the wife in the vignette. Under this theme, there were 86 participants blaming the victim for being anxious, social disconnectedness or lazy; and there were 166 participants blaming to the perpetrator being abusive, irresponsibility, lack of understanding, and cheating. There were 44 women believed the cause was relational, in which there were 41 participants attributed the problem to the broken relationship between the couple and three participants attributed to the lack of support. There were 28 participants believed the cause was communal and societal, such as being poor, family problems, fate, and believed IPV was a common scene. There were 15 participants could not identify the cause of IPV. These participants usually provided very brief responses and barely had insight on violent behaviors or confidence in discussing the cause. Our findings offer a direction for understanding the rural Chinese women's beliefs about the etiology of IPV to better develop interventions which must consider raising a public awareness campaign about the risk factors of IPV and focus on reducing self-blame among victims.

## Introduction

This study of intimate partner violence (IPV) in China occurs against an international backdrop of service developments for victims during the past half-century. IPV against women—a form of gender-based violence committed by a person who has a close personal relationship (typically, spousal or partner)—includes acts or threats of violence that result in harm or potential harm that is physical, sexual, or mental (1). According to the WHO (2), the lifetime prevalence of this major public health problem ranges from 13 to 61% globally for women; despite being a fundamental violation of human rights, it often has been ignored internationally despite decades of calls for greater recognition of the health and social needs of women.

**Figure 1:**
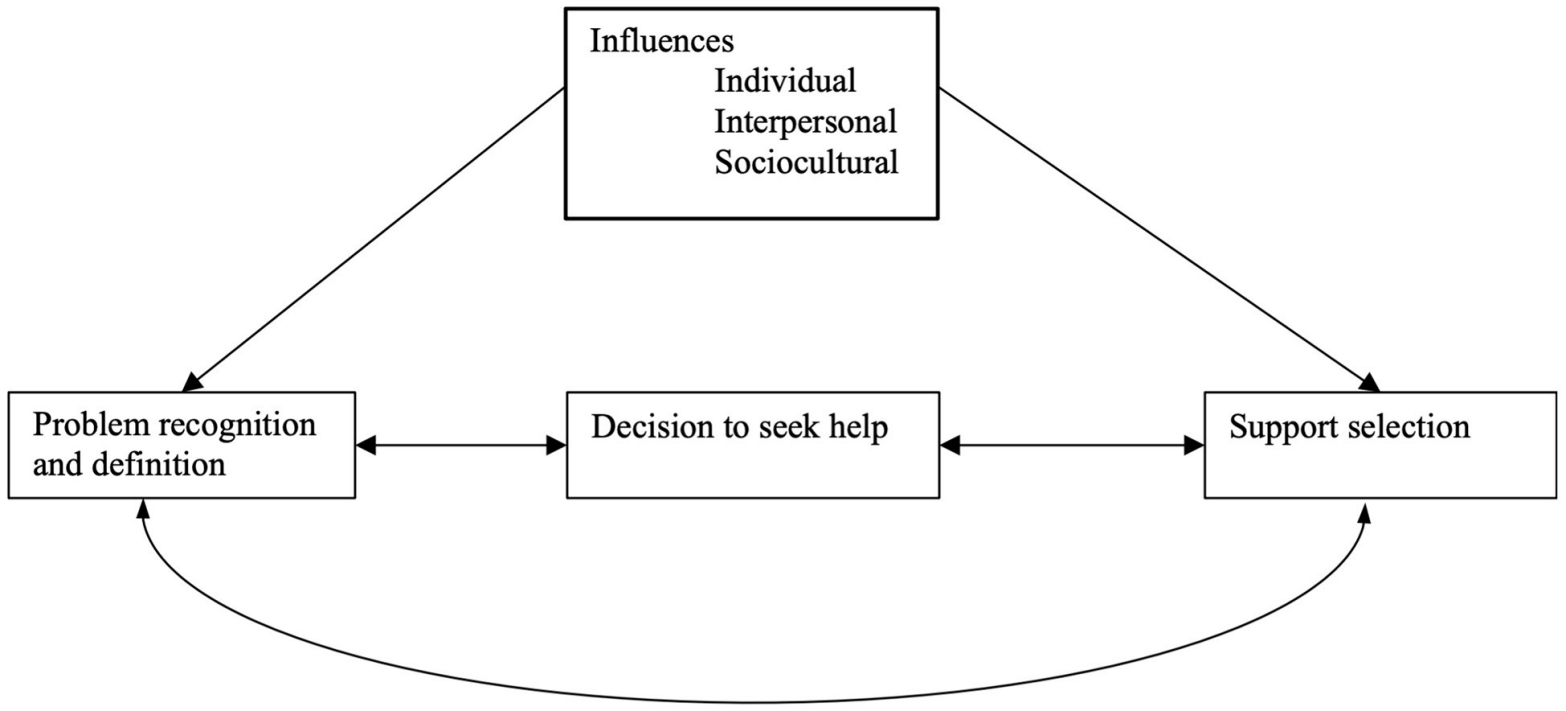
Liang's help-seeking framework for intimate partner violence victims.

It is in this context that some countries have taken the lead to develop needed resources for victims (3). During the 1970s, we saw the emergence of shelters and hotlines (4). In the ensuing years, programs offering social support and court advocacy developed (5, 6). In some nations, current services range from shelters to ongoing support, including provision of physical and mental health services (7). Recently, there have been initiatives to create medicine–law partnerships that offer coordinated, comprehensive services for victims at a single site, including the emergence of Family Justice Centers (8). However, despite all these services, many victims never seek help for myriad reasons. Liang et al. provided a theoretical framework to explain how victims make help-seeking decisions in the midst of their chaotic lives, and they suggested that individual, interpersonal, and community level variables influence help-seeking behaviors (Figure 1) (9). Additionally, abusive partners may control their victims in many ways that can interfere with help-seeking. Efforts to sabotage help-seeking can include threats and intimidation, forced coercion, and alternatively, promises to improve behavior and pledges of love and commitment. Liang et al. underscored that, while services may exist, survivors of abuse may not know how to seek them, in addition to fearing the repercussions from their partner if they do. Additionally, asking for help by reaching outside one's family may not be consistent with long-held cultural beliefs of perceived norms.

There have been relatively few studies of IPV in China, and these largely have been cross-sectional surveys designed to report the prevalence of IPV. In urban areas, Xu reported the incidence of physical violence ranged from 6 to 14% across age groups (10). Guo et al. reported that the lifetime prevalence of any IPV was 11.7% among pregnant women, aged 19–45 years (11). Another study among young women seeking abortions in hospitals found a lifetime prevalence of 22.6% (12).

Studies from rural areas typically found higher lifetime prevalence rates. These ranged from 19 to 29.7% for women suffering physical violence (13–15). Studies found prevalence as high as 58.1% among women suffering lifetime psychological violence and 16.7% for those experiencing lifetime sexual violence (14, 15). Other studies of IPV in China focused on exploring women's knowledge and attitudes of IPV with quantitative surveys. For example, 82.0 to 94.1% of urban residents identified physical violence such as hitting, kicking, and attacking with a weapon, yet only 16.1 to 20.8% recognized teasing, cursing, or deliberate silence between partners as potentially abusive behaviors (16). Further, it was also reported that women would justify violence for causes such as disobedience to parents-in-law, refusing to have sex with one's husband, and having affairs (17).

It is likely that traditional Confucian culture may have an especially strong influence on women living in rural Chinese communities who experience IPV, where residents are less likely to experience Western culture influences. Consequently, rural Chinese women may believe they are inferior, in turn diminishing help-seeking. A study involving married female migrant workers, most of whom came from rural China, reported that 11.1% agreed that a husband could beat his wife when she showed no or little filial respect for parents-in-law, and 15.7% agreed a husband could beat his wife when she had another sexual partner (17). Some Chinese cultural beliefs do not encourage victims to seek help from others; e.g., the traditional Chinese value “*jia chou bu ke wai yang*” (“domestic shame should not be made public”) may stop rural Chinese victims from seeking help due to shame and stigma. Further, as a “high context culture” that values indirect communications that are interpreted contextually, individuals may repress expressions of personal need, of “self,” to avoid conflicts and achieve harmony (18).

The purpose of this study was to qualitatively study rural Chinese women's perspectives on what causes IPV in the context of their lived experiences. To discern the perspectives of our rural participants, we used a mixed-method semi-structured interview to begin to piece together how a social ecological model (SEM) might inform future interventions.

## Methods

### Sample and Sampling

We drew our sample from a larger epidemiological study that assessed mental health problems and associated factors among rural women of Guangyuan City (19, 20). Guangyuan City was one of the most economically underdeveloped regions in Sichuan province (21). With the assistance of local health providers, the parent study used multi-stage sampling to recruit a random socio-economically diverse sample of 13 villages. To be included, women needed to be 16 years of age and older (16 is the age of consent in China), to have been living locally for at least 2 years, and to have reported they were married or in a relationship in the preceding year. Potential participants who carried a diagnosis of a major psychiatric disorder (e.g., schizophrenia and intellectual disability), based on registration lists provided by local hospitals, were excluded. All study participants provided oral consent.

The qualitative study explores the phenomena of IPV, and we applied probabilistic sampling to include every fifth woman who reported they were married or in a relationship in the preceding year from the parent study's sample (22). We compensated participants for their time with toiletry items (such as toothpaste and soap) worth 5 yuan (about 0.8 USD).

### Ethical Statement

The Ethics Committee of Sichuan University reviewed and approved the protocol, including the verbal informed consent process. The University of Rochester Research Subjects Review Board reviewed the approval from Sichuan University and approved analyses of de-identified data. Local health authorities from Guangyuan City assisted with the overall implementation of the study, and local health providers aided recruitment of participants.

### Measures

#### Demographic Information Questionnaire

We collected sociodemographic information including age, education, occupation, family structure including nuclear family (a pair of adults living with their children), stem family (the grandparents, their married children, and grandchildren who live together under the authority of the grandparents), joint/expanded family (more than two related nuclear and/or stem families living in a single household), partner's occupational status (migrant work or not), and family annual income.

## Short Form of the Revised Conflict Tactics Scale

We used the Short Form of the Revised Conflict Tactics Scale (CTS2S) to assess participants' current violence experiences, which has been widely applied globally (23–25); the Chinese version was translated in Hong Kong and has been applied in the Mainland with good reliability and validity (26–28). It contains statements about IPV experiences, and each statement examines the frequencies of IPV experiences from “zero” to “more than 20 times” during the past 12 months. We administered six victimization statements across three domains of IPV and coded any of the endorsed statements as a positive screen for IPV. Cronbach's α was 0.85 for this study sample.

## Short Explanatory Model Interview

To assess participants' explanatory models (EMs) of IPV, we applied the Short Explanatory Model Interview (SEMI), a brief validated assessment based on the EMs (29–31). The EMs are how people understand diseases, relate meaning to symptoms, explore the causalities, and express their expectations of treatment and related outcomes, which are culturally determined.

The SEMI explores details related to EMs of disease and helps researchers understand the perceived cause of the condition, the timing and mode of onset of the symptoms, the perceived pathophysiological processes involved, the beliefs about the natural history and severity of the condition, and the appropriate treatments for the condition. There are six sections (Health & Illness, Perceived Severity, Expectation & Satisfaction, Activities & Functioning, Other Health Behaviors, and Vignettes), and each section consists of a series of open-ended questions and probes in plain language without any professional medical terms. The SEMI encourages participants to provide answers based on their own experiences, attitudes, and beliefs to explore the relationship among research questions, social/natural circumstances, and social/cultural circumstances.

This study focused on the vignette section of the SEMI. Vignettes are brief narratives about hypothetical situations that are often used in qualitative research to elicit information about how a respondent thinks about a situation. They are particularly helpful for eliciting information about sensitive topics that respondents might not feel comfortable discussing about themselves (32). The vignette used was developed to depict an IPV situation in rural China (see below); we read the vignette to participants and then asked them to respond to open-ended questions related to the EM. An interdisciplinary team wrote the vignette: a psychiatric doctor, a primary care physician, and an attorney with IPV intervention experience, in partnership with two epidemiologists with rich epidemiological research experience in rural China.

*There is a woman, 35 years old and doing agriculture work. Her husband is a migrant worker in a city far away and barely comes home. Recently, her husband returned home from the city for a visit, but he kept yelling at her, calling her names. He said she embarrassed him in front of his friends because she wasn't well educated. Now, he barely talks to her at home, and he beats her when he is drunk or loses money after gambling. Every morning, compared to getting up and then doing work, she prefers to stay in bed and cover herself under the comforter. She feels so lonely, and she misses her family very much*.*Question: What are the causes of her problem?*

### Data Collection and Quality Control

In July 2012, we conducted the interviews with recruitment help from the local government and Guangyuan Mental Health Center as coordinators. We implemented strategies to increase the trustworthiness of the study. First, coordinators contacted village leaders and village doctors and held public information sessions about the study in villages before the survey began. Second, during the field survey, village leaders, doctors, and seniors escorted study interviewers door-to-door to conduct surveys for the larger study to increase participants' trust in this study; as some villages have low population density, local residents helped interviewers by transporting them with motorcycles. Third, considering IPV as a sensitive topic, we conducted the qualitative interview after participants completed the quantitative questionnaire for the larger study; and we conducted the interviews in private settings without any disturbance from participants' family members, neighbors, and friends. Interviewers recorded participants' responses to questions. We also randomly audio-recorded 46 interviews after participants' oral consent at the beginning of the field survey for quality control. Fourth, to reduce misunderstandings during interviews, we recruited graduated students who could speak and understand the local dialect in Guangyuan City as interviewers. The interviewers completed training sessions related to semi-structured interview methods and skills. There were three research teams, and each team had eight interviewers led by experienced senior researchers. We required all interviewers to check for missing items in the questionnaire after each interview, and they would complete missing items before leaving the household. Senior researchers reviewed recorded interviews, discussed encountered challenges and difficulties, developed strategies to improve interviewers' skills, and conducted quality control meetings to address issues.

### Coding and Analysis

**Figure 2:**
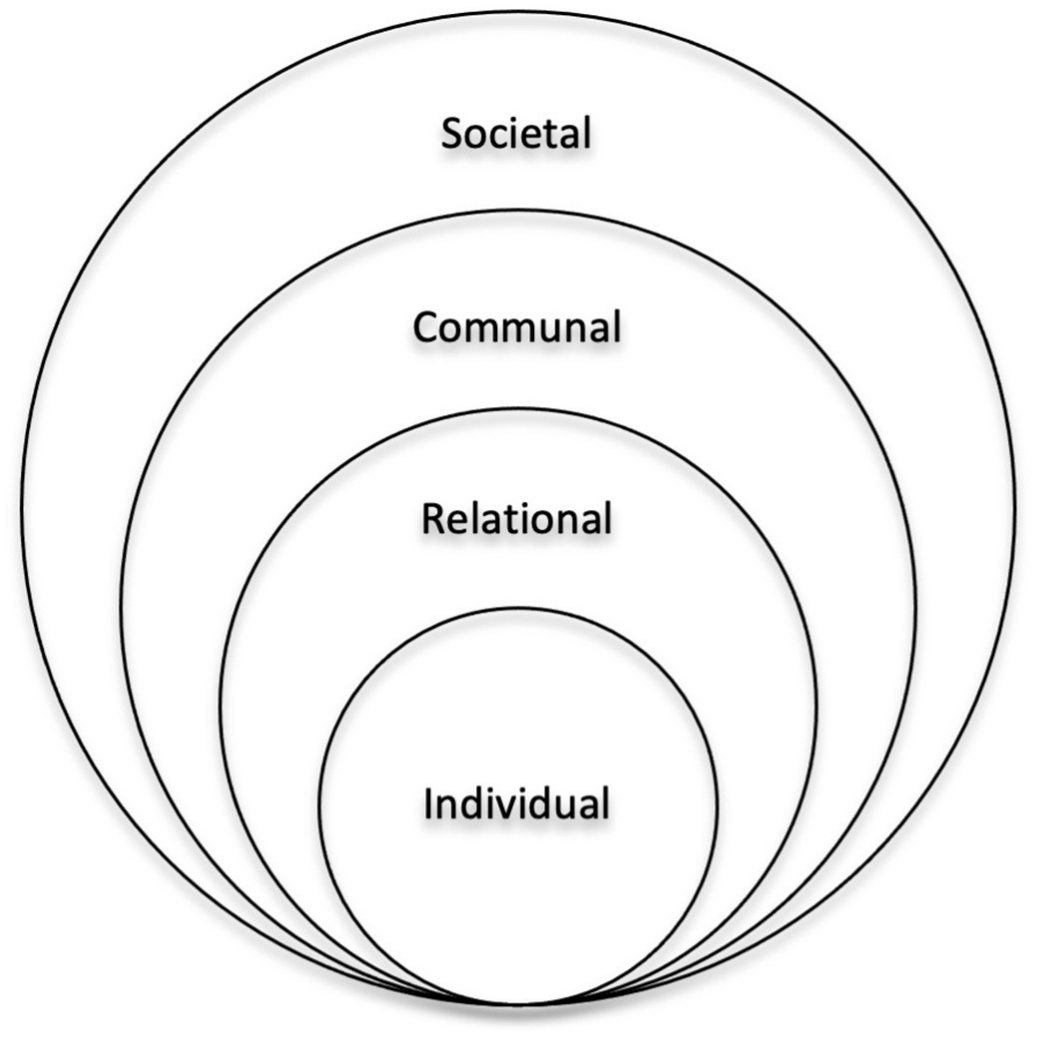
The social ecological model for understanding the cause of intimate partner violence.

International studies reveal common risk factors of IPV, and a helpful lens to view these risk factors/causes is the social ecological model (SEM) (33, 34). According to the model, IPV results from the interactions of associated factors that are embedded at individual, relational, communal, and societal levels and the overlapping rings in the model illustrate how factors at one level can influence factors at another level (Figure 2).

We applied the SEM to explore and understand rural women's explanations of IPV (33). Because this was a heavily scripted interview with a vignette prompt, we did not use the grounded theory. Rather, we read through all responses, selected the SEM as a framework, and utilized this approach (35). Thus, we could avoid coding data that were heavily influenced by the interview prompts. There were four coders who independently coded the qualitative data into categorical and numerical codes based on the SEM. If their codes were different for the same passage, the team would discuss the discrepancy until reaching consensus. After coding, we entered the categorical and numerical data into a database for content analysis.

## Results

We selected a sample size of 379 participants out of 1,898 participants from the larger study, and 339 of them (89.45%) completed the semi-structured interview.

Participants were 19 to 81 years old with 57.23% (194/339) between 40 and 59 years old, and the mean age was 46.01 years (SD: 12.42); 99.71% (338/339) were of Han ethnicity (Han is the dominant ethnic group in China); 31.86% (108/339) had never been educated, and 44.25% (150/339) had received <6 years of education (primary school); 50.44% (171/339) were living in a stem family; 14.75% (50/339) had migrant worker partners. Of participants' family annual income, 28.32% (96/339) and 23.00% (78/339) ranged from 10,000 to 19,999 yuan and 20,000 to 29,999 Yuan, respectively; of note, in 2012, the low, lower-middle, middle, upper-middle, and high-income households in rural Sichuan were defined as per capita annual income under 3,074, under 4,950, 6,709, 8,90,5 and 14,428 Yuan (36) (see Table 1 for details).

**Table 1:** Participants' characteristics and IPV experiences (*N* = 339).

**Demographic information**		**Any IPV**		**Frequency**	**Proportion**
		**experience**			**(%)**
		**No**	**Yes**		
Age (years old)	19–29	26	19	45	13.27
	30–39	30	23	53	15.63
	40–49	49	62	111	32.74
	50–59	43	40	83	24.48
	60 and above	24	23	47	13.86
Ethnicity	Han	171	167	338	99.71%
	Others	1	0	1	0.29%
Education	No school	58	50	108	31.86
	Less than 6 years	73	77	150	44.25
	More than 7 years	41	40	81	13.89
Family type	Living along/with friends	4	5	9	2.65
	Nuclear family	70	70	140	41.30
	Stem family	85	86	171	50.44
	Extended family	13	6	19	5.60
Husband as	No	144	136	280	82.60
migrant worker	Yes	24	26	50	14.75
	Unmarried	3	4	7	2.06
Annual family	0–9,999	29	34	63	18.59
income (Yuan)	10,000–19,999	47	49	96	28.32
	20,000–29,999	40	38	78	23.01
	30,000–39,999	23	16	39	11.50
	40,000–49,999	15	13	28	8.26
	50,000 and above	18	17	35	10.32
Total		172	167	339	

Overall, 49.26% (167/339) of participants had experienced violence from their partners in the last 12 months. The rate of physical, psychological, and sexual violence was 25.07% (85/338), 46.61% (158/339), and 7/08% (24/339), respectively.

### Explanations of Intimate Partner Violence

Overall, the participants' responses to the vignette question “What are the causes of her problem?” fell into four main themes related to their perceptions of the cause of IPV in the vignette: individual factors (*n* = 225), relational factors (*n* = 44), communal and societal factors (*n* = 15), and unidentified (*n* = 15). We briefly presented these themes in Table 2 and describe them below.

**Table 2:** The causes of IPV in the vignette explained by participants (*N* = 339).

**Causes of IPV**	***N***	**Examples of answers**
Individual factors	225	•“I think the cause is she has too much pressure.” •“It is because everything goes against her, and she has pressure.” •“Her husband works hard for the family, she doesn't care about him, of course he is angry.” •“She doesn't do housework at all, that's how she pisses her husband off.” •“He drinks, gambles and beats her.”
Relational factors	44	“They have problems in communicating with each other.” “They have been separated too long, and the relationship goes down.” “…either one of them cheated the other…”
Communal and societal factors	15	•“Her family doesn't have much money, they are poor.” •“Financial issues I guess, her family doesn't have much money to live well, nor does her.” •“The family financial problem makes them suspicious to each other.” •“It's her fate, she doesn't have a good husband, and she can't have a good life in the future.”
Unidentified	15	•“I don't know.” •“It's all about the young generation, I'm old and don't know about these things for a whole life.”

## Individual Factors

The majority of the responses (*n* = 225) fell into a theme we termed “individual factors,” which suggested participants attributed the conflicts in the vignette to either the woman or the man. We created profiles for the descriptions of the women that fell into this theme. Please see Table 3 for an example. These participants primarily discussed the social characteristics, behaviors, personalities, or even health problems of the husband or the wife in the vignette.

**Table 3:** The causes of IPV at individual level explained by participants.

**Individual causes**	***N***	**Subcategory of factors**	**Examples of answers**
Victim blaming	45	Mental problems	•“I think the cause is she has too much pressure.” •“Her unhappiness is in the Sixiang (mind or thoughts).” •“It is because everything goes against her, and she has pressure.” •“She is in a bad mood and hasn't been relieved from the feelings yet.” •“She has few contacts with the outside world, plus her husband has left for a long time, she has some kind of anxiety.” •“She is looking for death.”
	22	Lack of interpersonal communication skills, no education or inabilities	•“She does this to herself.” •“The reason is she spoils her husband.” •“She definitely has a bad attitude when she talks to her husband.” •“She doesn't do housework at all, that's how she pisses her husband off.” •“She is not independent and lazy.” •“She has no education, if not, her husband won't act like this.” •“Her husband works hard for the family, she doesn't care about him, of course he is angry.” •“She has never been in school, she can't do things other educated people can, no wonder her husband is mad about her.”
	19	Bad characters	•“The reason is she is not confidant.” •“Her weakness results in this.” •“She is introvert.” •“She grows a habit as being in bed.”
Perpetrator blaming	29	Torturing	•“He tortures her.” •“It is domestic violence.” •“He drinks, gambles and beats her.”
	77	Being irresponsible	•“If she has already taken care of him so much and he still acts like this, his problem, he shouldn't (do this to her).” •“…he doesn't care about her at all.”
	8	Verbal abuse	•“…he curses her on purpose.”, •“He talks about her shortcomings and irritates her.” •“He gets drunk and loses in gambling, so he takes it all on her.”
	12	Lack of understanding	•“I blame her husband for not understanding her.” •“Her husband works away from home, she does everything at home, but when he comes back, he still doesn't understand her contribution to the family.” •“He suspects she cheats.”
	18	Despise	•“…he is ashamed of her no education experience.” •“I blame him. He knows she is not well educated when he met her, but he still married her. Now he blames her for being not educated and he is afraid of losing his face.” •“He has saved some money when he works in the city, and she doesn't make any money, so that he despises her.” •“The cause is he has money, men always turn bad once they have money, he begins to look down upon her.”
	16	Cheating	•“He has affairs, so he treats her bad.” •“She is too naïve, her husband cheats on her and bullies her.” •“He has changed when he works outside.” •“Her husband fools around with women outside.”
	6	Disappointment	•“He is gambling all the time and doesn't listen to his wife nor any goals in life.” •“This husband is not a useful man, and he doesn't care the family.” •“He hangs around and does nothing and fails his responsibility.”

Over one-third of participants (*n* = 86) described the cause as the woman was “anxious,” “social disconnectedness,” “lazy,” or “introvert,” which we interpreted as victim blaming. A common term merged in this theme: mental problems. This umbrella term referred to a series of psychological or psychiatric problems. For example, “She has few contacts with the outside world, and her husband has left for a long time. “So she has some kind of anxiety,” and “Her unhappiness is in the Sixiang (which can be roughly translated as mind or thoughts),” and even “She is looking for death.” At other times, the participants associated the conflict with the woman's characteristics or skills. One participant stated, “She has never been educated in school, and she can't do things like other educated people can. No wonder her husband is mad at her,” while another said, “She doesn't do housework at all, that's how she pisses off her husband.”

The remaining participants in this category (*n* = 166) attributed blame to the husband and described the cause of the man as “torturing,” “being irresponsible,” “verbal abuse,” “lack of understanding,” “despise,” “cheating,” and “disappointment,” which we interpreted as perpetrator blaming. A common theme that surfaced among responses was irresponsibility. For example, “If she has already taken care of him so much and he still acts like this, his problem, he shouldn't (do this to her),” and “…he doesn't care about her at all.” Some participants directly recognized violence or pointed out the term “domestic violence” in simple responses, such as “He tortures her” and “It's domestic violence.” There were also participants who associated the conflict to how the man defended his face (“face” referred to the dignity or prestige an individual processes in Chinese culture). One participant stated, “I blame him. He knows she is not well-educated when he met her, but he still married her. Now he blames her for being not educated and he is afraid of losing his face.”

## Relational Factors

There were 44 participants who believed that the cause was relational. Forty-one women attributed the problem to the broken relationship between the couple, and examples were “They have been separated too long, and the relationship goes down,” “He works far away, now they don't fit each other,” “They have problems in communicating with each other,” “It's mutual, they don't listen to or understand each other,” “… either one of them cheated the other,” and “…they have too many conflicts.” Three women attributed the problem to her lack of support, and their responses were “She can only get comfort when she moves back to her parents,” “She is alone at home, no one looks after her and no one understands her,” and “She needs support.”

## Communal and Societal Factors

There were seven women who believed that the cause was community factors, and their responses were “Her family doesn't have much money, they are poor,” “I think it's her living environment,” “It's her family,” “She is upset about family problems,” “Financial issues I guess, her family doesn't have much money to live well, nor does her,” “They both work too hard and feel very tired, he drinks, gambles and she wants to stay in bed,” and “The family financial problem makes them suspicious to each other.”

There were 21 women who believed the cause was societal factors. Two women believed the cause was her fate, and their responses were “It's her fate, she doesn't have a good husband, and she can't have a good life in the future,” and “It's fate that made her born in the rural areas, so that she didn't have a chance to go to school.” Nineteen women believed it was a common/normal scene in life and that there was no cause for anything, and examples were “…the couple quarrels, how common it is,” “No reason, no cause, there is nothing behind it,” and “I don't think there is any problem or cause.”

## Unidentified

Few participants had responses (*n* = 15) that fell into the theme that we termed “unidentified.” These participants usually provided very brief responses and appeared to barely have insight on violent behaviors or confidence in discussing the cause. Common responses were “I don't know” or “I can't think about it.” Despite careful probing by interviewers, these participants had little to say; they may have lacked the language to discuss the story. For example, one woman stated, “It's all about the young generation. I'm old and don't know about these things for a whole life.”

## Discussion

To our knowledge, this is the first qualitative study in China to elicit the perspectives—to hear the voice—of rural Chinese women and to explore how they understand the cause of IPV. This study fills a gap in the literature and provides findings to develop IPV interventions in rural China. We reported 15 participants who provided very brief responses and appeared to barely have insight on violent behaviors or confidence in discussing the cause; 252 participants attributed the conflicts to individual problems; 44 women believed the cause was relational; and 28 participants attributed the cause of IPV to communal and societal factors.

The WHO suggests the SEM when considering global violence as well. IPV studies have documented individual level risk factors to include younger age (37), lower level of education (38), financial dependence (39), previous violence experience (40), lower level of empowerment and lower social support (41), and childhood maltreatment experience (42). At the relational level, risk factors include multiple partners (43), male dominance in the family (44, 45), economic stress (34), poor family functioning and low social capital (46), and in-law conflicts (47). At the communal level, risk factors include poverty (48) and weak community sanctions (46). At the societal level, risk factors include traditional gender and social norms, which support violence (33, 49, 50), ideologies of male entitlement (51), and weak legal sanctions (34).

We sought to understand rural women's explanations to IPV based on the SEM as a framework, and we learned that the gender inequality in the traditional Chinese culture may have sculpted participants' perspectives. IPV reflects the relationship power, and the more dominant males are, the more females will experience violence, regardless of countries/regions (52, 53). Confucianism deeply influences the Chinese traditional culture, which has been criticized for gender norms that belittle females, e.g., the lifetime doctrine, “*san cong si de*” (the three obediences that women should obey their fathers, serve their husbands' need, and follow their sons; and the four virtues that include fidelity, tidiness, propriety in speech, and commitment to needlework) (50). The traditional Chinese gender concepts advocate a husband should be dominant, strong, and arbitrary in the family and within his relationship. On the other hand, a woman should be submissive to her husband and other male family members and obedient in her relationship (54). Traditional virtues such as “*san cong si de*” and “*san gang wu chang*” (the three rules and five constant virtues of Confucianism; those are ruler guides subject, father guides son and husband guides wife, benevolence, righteousness, propriety, wisdom, and fidelity) and “*nan zhu wai, nv zhu nei*” (men are responsible for outdoor affairs; women are responsible for indoor affairs) exactly reflect this inequality. Meanwhile, men who have attitudes that beating wives is acceptable are two times more likely to commit IPV than those who do not (42). Given this cultural background, rural women are very dependent on men. When conflicts arise, women often feel guilty and will not adopt appropriate coping strategies to reduce violence and protect their safety (55, 56). Hence, mitigating the patriarchal influences of traditional rural Chinese concepts may be an important first step for interventions to encourage help-seeking behaviors to reduce IPV.

When participants attributed IPV to relational problems, we believed it was related to the internal migration in China. Along with China's economic development, the population of internal migrant workers, who were mainly males, increased. Consequently, the population of those left behind, mainly women (predominantly married), children, and elders in rural areas, increased (57–61). The separation of couples may reduce communication and mutual understanding due to the differences in urban and rural living experiences that would gradually increase the gap in how to cope with daily stress and solve relationship conflicts. Participants relayed that this may increase the risk of IPV in return. Chen's study with a rural Chinese sample reported that the main causes for IPV were financial conflicts, gambling, substances abuse (smoking, drinking and drug abuse), personality conflicts, affairs, sex experience, and reproductive problems (62); further, the study reported women listed financial conflicts and men listed cheating as the primary cause of IPV, which raised a question worthy of further discussion and exploration. Why did women focus on financial conflicts and men focus on affairs? Chen's study indicated the rural-to-urban internal migration was at the societal level, and interventions should address its influence on IPV.

We need to consider the limitations of this study. First, we only implemented home interviews during daytime hours, thus missing women who had outside work, and we did not recruit female migrant workers living in distant cities. Consequently, we did not obtain the perspectives of women who may have been more independent. Second, though the vignette component of the SEMI has been shown to be useful in understanding participants' beliefs about diseases states and EMs of illnesses, it is possible that we would have elicited different responses had we asked the participants to describe their own experiences with IPV or to imagine themselves in the scenario described (63). Third, we coded participants' main responses in the analysis. While we did not record data to explore the inter-rater reliability of coding, we did use consensus. Nevertheless, this study's findings are an important first step, despite its limitations, in beginning to craft culturally appropriate IPV interventions in rural China.

## Conclusion

Despite the limitations, this is the first study that we are aware of that sought to hear rural women's voices about the causes of IPV. It is imperative to assess community members' perceptions of what causes IPV to create interventions to prevent and address this public health issues. We report that a majority of rural women attribute IPV to the victim's or perpetrator's personal problems, indicating that IPV interventions must consider raising a public awareness campaign about the risk factors of IPV and focus on reducing self-blame among victims. Liang's theory of help-seeking suggests that victims' decisions on whether and where to seek help are influenced by their living experiences, interpersonal social support system, and sociocultural norms; and these factors will also impact how victims identify IPV. China is rooted in traditional heteronormative cultural beliefs that women are subordinate to men. Our findings help in understanding the rural Chinese women's beliefs about the etiology of IPV, and we believe that to overcome cultural barriers and promote help-seeking behaviors, interventions should target cultural factors from each level of the SEM.

Fundamentally, we are concerned that many rural victims' experiences remain hidden due to barriers at multiple levels—cultural as well as lack of infrastructure (e.g., IPV shelters), lack of information about crisis call lines, lack of transportation to seek help, and limited access to the legal and justice system. In this context, strategies to reduce the prevalence and impact of IPV in rural China should focus both on improving communities' capacities to prevent and respond to IPV and on disseminating processes that encourage help-seeking behaviors and fostering a new generation of social norms (64). These must be culturally attuned and adapted to the needs of China and implemented locally to have sustained impact.

## Data Availability Statement

The raw data supporting the conclusions of this article will be made available by the authors, without undue reservation.

## Ethics Statement

The studies involving human participants were reviewed and approved by the Ethics Committee of Sichuan University and the University of Rochester Research Subjects Review Broad. Written informed consent for participation was not required for this study in accordance with the national legislation and the institutional requirements.

## Author Contributions

FH designed the study instruments, implemented the field study, monitored data collection, cleaned the data, developed the plan for analysis, analyzed the data, and drafted and revised the paper. CC designed the study instruments, trained interviewers, assisted with the analysis plan, revised the paper, and supervised FH. MW designed the study instruments, trained interviews, and revised the paper. EC initiated the project, revised the paper, and supervised FH. PQ initiated the project, designed the study instruments, monitored data collection, cleaned the data, and revised the paper. All authors had full access to all the data in the study and take responsibility for the integrity of the data and the accuracy of the data analysis. All authors read and approved the final manuscript.

## Funding

This research was supported, in part, by NIH Grant D43 TW009101 and TW009101-01S1 (EC, PQ).

## Conflict of Interest

The authors declare that the research was conducted in the absence of any commercial or financial relationships that could be construed as a potential conflict of interest.

## Publisher's Note

All claims expressed in this article are solely those of the authors and do not necessarily represent those of their affiliated organizations, or those of the publisher, the editors and the reviewers. Any product that may be evaluated in this article, or claim that may be made by its manufacturer, is not guaranteed or endorsed by the publisher.

## References

[1] United Nations General Assembly,. Declaration on the Elimination of Violence Against Women. (1993). Available online at: https://www.un.org/en/genocideprevention/documents/atrocity-crimes/Doc.21_declaration%20elimination%20vaw.pdf (accessed Feburary 23, 2013).

[2] World Health Organization. Violence Against Women: Intimate Partner and Sexual Violence Against Women. (2013). Available online at: http mediacentre/factsheets/fs239/en/ (accessed Janurary 17, 2014).

[3] Eckhardt CI Murphy CM Whitaker DJ Sprunger J Dykstra R Woodard K. The effectiveness of intervention programs for perpetrators and victims of intimate partner violence. Partner Abuse. (2013) 4:196–231. 10.1891/1946-6560.4.2.19625528978

[4] Berk RA Newton PJ Berk SF. What a difference a day makes: an empirical study of the impact of shelters for battered women. J Marriage Fam. (1986) 48:481–90. 10.2307/352034

[5] Kocot T Goodman L. The roles of coping and social support in battered women's mental health. Viol Against Women. (2003) 9:323–46. 10.1177/107780120225007529294903

[6] Camacho CM Alarid LF. The significance of the victim advocate for domestic violence victims in municipal court. Viol Vict. (2008) 23:288–300. 10.1891/0886-6708.23.3.288 18624095

[7] New M Berliner L. Mental health service utilization by victims of crime. J Traum Stress. (2000) 13:693–707. 10.1023/A:1007818402277 11109240

[8] Simmons CA Howell KH Duke MR Beck JG. Enhancing the impact of Family Justice Centers via motivational interviewing: an integrated review. Trauma Viol Abuse. (2016) 17:532–41. 10.1177/1524838015585312 25966969

[9] Liang B Goodman L Tummala-Narra P Weintraub S. A theoretical framework for understanding help-seeking processes among survivors of intimate partner violence. Am J Commun Psychol. (2005) 36:71–84. 10.1007/s10464-005-6233-6 16134045

[10] Xu X. The prevalence and determination of wife abuse in urban China. J Comp Fam Stud. (1997) 28:280.25104345

[11] Guo S Wu J Qu C Yan R. Physical and sexual abuse of women before, during, after pregnancy. Int J Gynecol Obstetr. (2004) 84:281–6. 10.1016/j.ijgo.2003.08.019 15001384

[12] Wu J Guo S Qu C. Domestic violence against women seeking induced abortion in China. Contraception. (2005) 72:117–21. 10.1016/j.contraception.2005.02.010 16022850

[13] Parish WL Wang T Laumann EO Pan S & Luo Y. Intimate partner violence in China: national prevalence, risk factors and associated health problems. Int Fam Plann Perspect. (2004) 30:174–81. 10.1363/3017404 15590383

[14] Zhao F Guo S Wang L Wu J Wang L. Investigation on the patterns and knowledge regarding domestic violence among married women in rural areas of China. Chin J Epidemiol. (2006) 27:664–8. 10.3760/j.issn:0254-6450.2006.08.006 17172104

[15] Guo S Zhao F Wu J Zhang T Wang L Wang L. Study on prevalence of domestic violence and its related factors in rural area of China. Chin J Public Health. (2007) 23:4–6. 10.11847/zgggws2007-23-01-03

[16] Sun S. The Study on congnition of family violence among population in Weihai (Doctoral dissertation). Shandong University, Shandong, China (2006).

[17] Sun F Lou C Cheng Y Tu X. Study on the attituders to domestic violence in those married migrant women. J Int Reprod Health Fam Plann. (2012) 31:178–81. 10.3969/j.issn.1674-1889.2012.03.007

[18] Kim D Pan Y Park HS. High-versus low-context culture: a comparison of Chinese, Korean, American cultures. Psychol Market. (1998) 15:507–21.

[19] Hou F Cerulli C Wittink M Caine E Qiu P. Depression, social support and associated factors among women living in rural China: a cross-sectional study. BMC Women's Health. (2015) 15:28. 10.1186/s12905-015-0180-7 25879808 PMC4392745

[20] Qiu P Caine E Hou F Cerulli C Wittink M Li J. The prevalence of distress and depression among women in rural Sichuan province *PLoS ONE*. (2016) 11:e0161097. 10.1371/journal.pone.0161097 27526182 PMC4985145

[21] Statistic Bureau of Sichuan Province China. Sichuan Province Statistics Yearbook. (2011). Available online at: http://tjj.sc.gov.cn/scstjj/c105855/2001/12/15/0286dab886a8457fbcb3798c27d42e18/ (accessed December 31, 2012).

[22] Liamputtong P. Researching the Vulnerable: A Guide to Sensitive Research Methods. Thousand Oaks, CA: Sage (2006).

[23] Straus MA Michel-Smith Y. Mutuality, severity, and chronicity of violence by father-only, mother-only, and mutually violent parents as reported by university students in 15 nations. Child Abuse Neglect. (2013) 38:664–76. 10.1016/j.chiabu.2013.10.004 24252745

[24] Mendoza J. The impact of minority stress on gay male partner abuse. In: Ristock JL editor. Intimate Partner Violence in LGBTQ Lives. Abingdon: Routledge (2011). p. 169–81.

[25] Woodcock KM. The Mental Health Help Seeking Experiences of Female Victims of Intimate Partner Violence. Pittsburgh, PA: University of Pittsburgh (2007).

[26] Chan KL. Correlates of wife assault in Hong Kong Chinese families. Viol Vict. (2004) 19:79–82. 10.1891/vivi.19.2.189.64104 15384454

[27] Straus MA Douglas EM. A short form of the Revised Conflict Tactics Scales, and typologies for severity and mutuality. Viol Vict. (2004) 19:507–20. 10.1891/vivi.19.5.507.63686 15844722

[28] Zhang N Gao Y Wu R Qiu P. Validity and reliability of the short form of the revised Conflict Tactics Scales in women suffering from domestic violence in rural areas. Chin Mental Health J. (2014) 28:381–384. 10.3969/j.issn.1000-6729.2014.05.011

[29] James SL Abate D Abate KH Abay SM Abbafati C Abbasi N. Global, regional, national incidence, prevalence, and years lived with disability for 354 diseases and injuries for 195 countries and territories, 1990-2017: a systematic analysis for the Global Burden of Disease Study 2017. Lancet. (2017) 392:1789–858. 10.1016/S0140-6736(18)32279-7 30496104 PMC6227754

[30] Kleinman A. Patients and Healers in the Context of Culture: an Exploration of the Borderland Between Anthropology, Medicine, Psychiatry (Vol. 3). Berkeley, CA: University of California Press (1980). 10.1525/9780520340848

[31] Lloyd KR Jacob K Patel V St Louis L Bhugra D Mann A. The development of the Short Explanatory Model Interview (SEMI) and its use among primary-care attenders with common mental disorders. Psychol Med. (1998) 28:1231–7. 10.1017/S0033291798007065 9794030

[32] Renold E. Using vignettes in qualitative research. Build Res Cap. (2002) 3:3–5.

[33] Krug EG Mercy JA Dahlberg LL Zwi AB. The world report on violence and health. Lancet. (2002) 360:1083–8. 10.1016/S0140-6736(02)11133-0 12384003

[34] Heise LL. Violence against women an integrated, ecological framework. Viol Against Women. (1998) 4:262–90. 10.1177/1077801298004003002 12296014

[35] Pope C Ziebland S Mays N. Analysing qualitative data. Brit Med J. (2000) 320:114. 10.1136/bmj.320.7227.114 10625273 PMC1117368

[36] Statistic Bureau of Sichuan Province China. Sichuan Province Statistics Yearbook 2013. (2013). Available online at: http://tjj.sc.gov.cn/scstjj/c105855/2001/12/17/573aaa5fa1c9423595dd9456723c19cb/ (accessed March 14, 2016).

[37] Harwell TS Spence MR. Population surveillance for physical violence among adult men and women, Montana 1998. Am J Prevent Med. (2000) 19:321–24. 10.1016/S0749-3797(00)00240-3 11064238

[38] Ackerson LK Kawachi I Barbeau EM Subramanian S. Effects of individual and proximate educational context on intimate partner violence: a population-based study of women in India. Am J Public Health. (2008) 98:507–14. 10.2105/AJPH.2007.113738 18235066 PMC2253590

[39] Chan KL. Study on Child Abuse and Spouse Battering: Report on Findings of Household Survey: a Consultancy Study Commissioned by the Social Welfare Department of the Hong Kong SAR. Hong Kong: University of Hong Kong (2005).

[40] Boyle MH Georgiades K Cullen J Racine Y. Community influences on intimate partner violence in India: Women's education, attitudes towards mistreatment and standards of living. Soc Sci Med. (2009) 69:691–7. 10.1016/j.socscimed.2009.06.039 19619925

[41] García-Moreno C Jansen HA Ellsberg M Heise L Watts C. WHO Multi-Country Study on Women's Health Domestic Violence Against Women: Initial Results on Prevalence, Health Outcomes Women's Responses. (2005). Available online at: https://apps.who.int/iris/bitstream/handle/10665/43309/924159358X_eng.pdf (accessed June 17, 2015).

[42] World Health Organization. Preventing Intimate Partner and Sexual Violence Against Women: Taking Action and Generating Evidence. (2010). Available online at: https://apps.who.int/iris/bitstream/handle/10665/44350/9789275716359_por.pdf. (acccessed June 17, 2015).

[43] Chan KL. Sexual violence against women and children in Chinese societies. Trauma Viol Abuse. (2009) 10:69–85. 10.1177/1524838008327260 19008337

[44] Tjaden P Thoennes N. Prevalence and consequences of male-to-female and female-to-male intimate partner violence as measured by the National Violence Against Women Survey. Viol Against Women. (2000) 6:142–61. 10.1177/10778010022181769

[45] Xu X Campbell JC Zhu F. Intimate partner violence against Chinese women-the past, present, and future. Trauma Viol Abuse. (2001) 2:296–315. 10.1177/1524838001002004002

[46] Heise L Garcia-Moreno C. Violence by intimate partners. In: Krug EG Dahlberg LL Mercy JA editors. World Report on Violence and Health. Geneva: World Health Organization (2002).

[47] Chan KL Tiwari A Fong DYT Leung WC Brownridge DA Ho PC. Correlates of in-law conflict and intimate partner violence against Chinese pregnant women in Hong Kong. J Int Viol. (2009) 24:97–110. 10.1177/0886260508315780 18378806

[48] Jewkes R Sen P Garcia-Moreno C. Chapter 6. Sexual Violence. World Report on Violence and Health. (2002). p. 147–81.

[49] Lisak D Hopper J Song P. Factors in the cycle of violence: gender rigidity and emotional constriction. J Traum Stress. (1996) 9:721–43. 10.1002/jts.2490090405 8902743

[50] Tang CSK Lai BPY. A review of empirical literature on the prevalence and risk markers of male-on-female intimate partner violence in contemporary China, 1987–2006. Aggress Viol Behav. (2008) 13:10–28. 10.1016/j.avb.2007.06.001

[51] Adams PJ Towns A Gavey N. Dominance and entitlement: the rhetoric men use to discuss their violence towards women. Dis Soc. (1995) 6:387–406. 10.1177/0957926595006003006

[52] Chan KL Liu T Tiwari A Leung WC Fong D Ho PC. Intimate partners' violence against Chinese pregnant women: a review of studies in manland China and Hong Kong. Collect Women's Stud. (2011) 2:87–94. 10.3969/j.issn.1004-2563.2011.02.012

[53] Levinson D. Family Violence in Cross-Cultural Perspective. Sage Publications, Inc. (1989). 10.1007/978-1-4757-5360-8_18

[54] Lin M He X. A study on Chinese women suffering from family violence. Shandong Soc Sci. (2010) 6:13.

[55] Bhuyan R Mell M Senturia K Sullivan M Shiu-Thornton S. “Women must endure according to their karma” Cambodian immigrant women talk about domestic violence. J Int Viol. (2005) 20:902–21. 10.1177/0886260505277675 15983130

[56] Boonzaier F. If the man says you must sit, then you must sit': the relational construction of woman abuse: gender, subjectivity and violence. Femin Psychol. (2008) 18:183–206. 10.1177/0959353507088266

[57] National Population Family Planning Commission of P.R.China. The 2012 Report on the Development of China's Migrant Population. (2012). Available online at: http://www.scio.gov.cn/zhzc/8/4/Document/1199460/1199460.htm (accessed January 20, 2016).

[58] Fan CC. Rural-urban migration and gender division of labor in transitional China. Int J Urban Reg Res. (2003) 27:24–47. 10.1111/1468-2427.00429

[59] De Brauw A Giles J. Migrant labor markets and the welfare of rural households in the developing world: evidence from China. World Bank Econ Rev. (2008) 32:1–18. 10.1093/wber/lhx023

[60] Mu R Van de Walle D. Left behind to farm? Women's labor re-allocation in rural China. Labour Econ. (2011) 18:S83–97. 10.1016/j.labeco.2011.01.009

[61] De Brauw A Li Q Liu C Rozelle S Zhang L. Feminization of agriculture in China? Myths surrounding women's participation in farming. China Q. (2008) 194:327–48. 10.1017/S030574100800040430886898

[62] Chen W. Study of somestic violence amang women in rural China. Stud Law Bus. (2007) 24:91–101.

[63] Dinos S Ascoli M Owiti J Bhui K. Assessing explanatory models and health beliefs: an essential but overlooked competency for clinicians. BJPsych Adv. (2017) 23:106–14. 10.1192/apt.bp.114.013680

[64] McDonnell KA Burke JG Gielen AC O'Campo P Weidl M. Women's perceptions of their community's social norms towards assisting women who have experienced intimate partner violence. J Urban Health. (2011) 88:240–53. 10.1007/s11524-011-9546-9 21336504 PMC3079036

